# Amazonian Discovery Sheds Light on the Evolution of *Caenocentron* Schmid, 1982 (Trichoptera: Xiphocentronidae): Phylogenetic Placement and Description of a New Species †

**DOI:** 10.3390/insects16121188

**Published:** 2025-11-22

**Authors:** Gleison R. Desidério, Albane Vilarino, Laissa da Silva dos Santos, Pitágoras C. Bispo, Neusa Hamada

**Affiliations:** 1 Laboratório de Biologia Aquática (LABIA), Programa de Pós-Graduação em Biociências—Interunidades, Faculdade de Ciências e Letras de Assis (FCLAs), Universidade Estadual Paulista “Júlio de Mesquita Filho” (UNESP), Assis 19806-900, Brazil; pitagoras.bispo@unesp.br; 2 Programa de Pós-Graduação em Biodiversidade Neotropical (PPGBN), Universidade Federal da Integração Latino-Americana (UNILA), Foz do Iguaçu 85870-650, Brazil; 3 Laboratório de Sistemática e Biogeografia de Insetos, Departamento de Zoologia, Instituto de Biociências, Universidade de São Paulo (USP), São Paulo 05508-900, Brazil; 4 Programa de Iniciação Científica (PIBIC), Instituto Nacional de Pesquisas da Amazônia (INPA), Manaus 69060-001, Brazil; ldsds.bio19@uea.edu.br; 5 Laboratório de Citotaxonomia e Insetos Aquáticos (LACIA), Programa de Pós-Graduação em Entomologia (PPGEnto), Instituto Nacional de Pesquisas da Amazônia (INPA), Manaus 69067-375, Brazil; neusaha@gmail.com

**Keywords:** aquatic insects, caddisflies, Xiphocentroninae, taxonomy, morphology, phylogeny, Amazon biome, Campos Amazônicos National Park

## Abstract

*Caenocentron* was previously hypothesized to have originated in Mesoamerica from Xiphocentronidae ancestors that dispersed from Asia to the Americas during Eocene hyperthermal events. Under this scenario, the genus underwent early diversification in Mesoamerica and reached South America only during the late Miocene, following the uplift and connection of Central America. However, the discovery of *Caenocentron roosevelt* sp. nov. in savanna areas of the Brazilian Amazon significantly revises this understanding. A morphology-based parsimony analysis of *Caenocentron* and related xiphocentronids (26 taxa, 46 characters) recovered *C. roosevelt* sp. nov. as the sister species to all other members of the genus. This placement implies that *Caenocentron* was in South America far earlier than previously inferred, likely by the late Oligocene. *Caenocentron roosevelt* sp. nov. is distinguished by the following combination of characters: the absence of a setose ventral projection on the coxopodite (present in all other congeners), the presence of apical lobes on tergum IX (reduced in other species), paired digitate lobes beneath the posterior margin of tergum IX (otherwise found only in one Nearctic species), and a distinct line of robust setae along the apical projection of the coxopodite (absent in other species). Additionally, a key to the males of all 10 currently recognized species of *Caenocentron* is provided.

## Simple Summary

Caddisflies are aquatic insects that play important roles in freshwater ecosystems and are useful for studying how species evolve and spread. The net-tube caddisfly genus *Caenocentron* (Xiphocentronidae) was previously thought to have originated in Central America and only reached South America millions of years later, after land connections formed between the continents. However, the discovery of a new species, *Caenocentron roosevelt*, in the savanna region of the Brazilian Amazon challenges this idea. Careful comparison of its morphological structures with related species shows that *C. roosevelt* sp. nov. is the earliest branch in the evolutionary tree of the group, suggesting that the genus has been present in South America much longer than previously believed—probably since the late Oligocene, about 25 million years ago. This new finding helps scientists better understand the ancient history and movement of insects between continents. The study also provides an identification guide to all known *Caenocentron* species, which will assist future research and biodiversity monitoring in South American freshwater ecosystems.

## 1. Introduction

Xiphocentronidae Ross, 1949 is a family of net-tube caddisflies comprising approximately 200 described species, predominantly distributed across tropical regions worldwide [1]. Adult members of this family are generally diurnal [2,3], while their larvae construct silken tunnels over wet rocks in splash zones outside of running water, where they graze on surface detritus and microalgae [4,5,6]. The family is currently divided into three subfamilies: Xiphocentroninae, which includes seven extant genera; Proxiphocentroninae, comprising a single Oriental genus [3]; and Palerasnitsyninae, containing only the fossil genus †*Palerasnitsynus*, recently transferred from Psychomyiidae to Xiphocentronidae [7].

In the New World, Xiphocentronidae is represented by three genera and 87 species, distributed from the southern United States to northern Argentina: *Caenocentron* Schmid, 1982, *Machairocentron* Schmid, 1982, and *Xiphocentron* Brauer, 1870. In recent years, the Neotropical fauna of Xiphocentronidae has received much attention, resulting in the description of a large number of species [1,8,9,10,11,12,13].

*Caenocentron* was originally proposed as a subgenus of *Cnodocentron* Schmid, 1982, encompassing species from the Americas and Southeast Asia [3]. However, a subsequent phylogenetic analysis demonstrated that *Caenocentron* is more closely related to other Neotropical genera than to the Oriental *Cnodocentron*, and it was elevated to genus level by Vilarino et al. [11]. This decision was supported by several synapomorphies: (1) basoventral margin of the coxopodite produced; (2) apical margin of the coxopodite produced posterad; (3) basal stout spine present on the harpago; (4) apex of the basal plate apodeme directed ventrally; and (5) basomesal setae of the coxopodite elongated [11].

**Figure 1:**
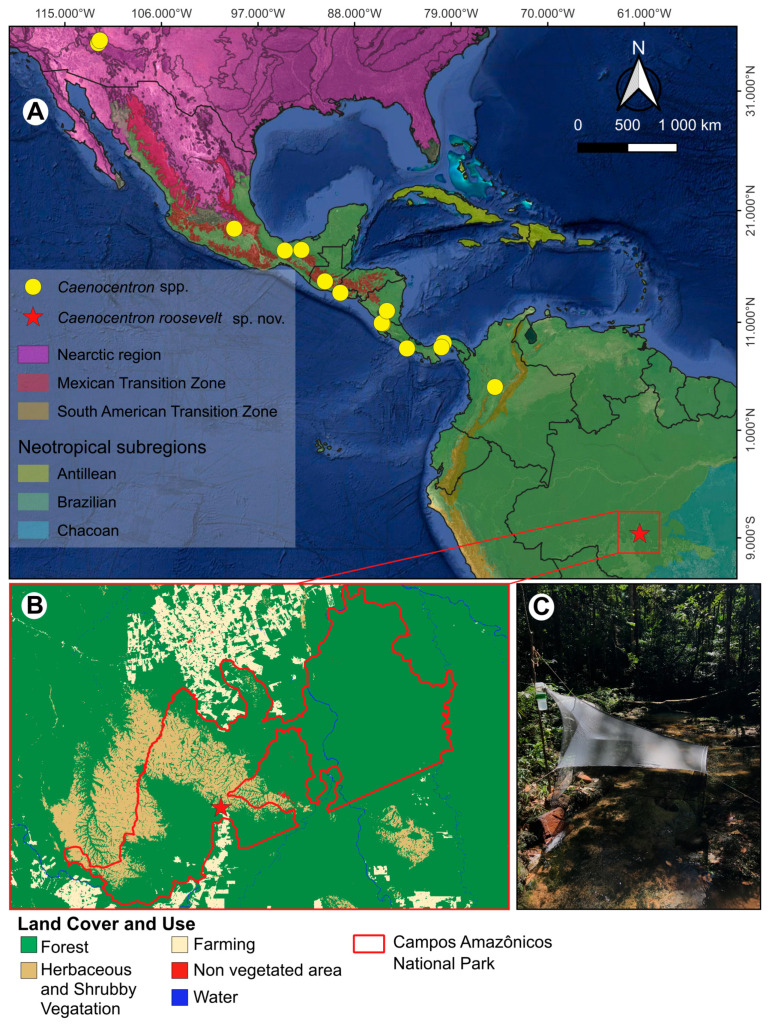
Distribution of species of *Caenocentron* Schmid, 1982 (Xiphocentronidae) in the Nearctic and Neotropical regions and general views of the collection site of *Caenocentron roosevelt* sp. nov.: (**A**) geographical distribution map for all *Caenocentron* species; (**B**) map of Campos Amazônicos National Park showing different land cover and use from MapBiomas Collection—2024, with indication of the type locality (red star) of *C. roosevelt* sp. nov.; (**C**) Onça stream, Campos Amazônicos National Park, Amazonas state, Brazil, type locality of *C. roosevelt* sp. nov.

Currently, *Caenocentron* includes nine described species, most of which are found in Mesoamerica and Central America. Only two species have been reported outside this biogeographical or continent region: *C. yavapai* Moulton & Stewart, 1997 from the southern United States (Nearctic region) and *C. immaculatum* Flint, 1991 from northern Colombia (South America) (Figure 1A). The time-calibrated phylogeny suggests that *Caenocentron* diverged from a non-monophyletic *Xiphocentron* during the late Eocene, approximately 37 Mya [11]. Biogeographical analyses [11] indicate that the genus diversified in association with the tectonic movement of the Chortis block, the formation of Central America, and the emergence of land connections to South America [14,15]. According to this reconstruction, *Caenocentron* reached South America only during the late Miocene, around 8 Mya [11].

While surveying caddisflies in the Brazilian Amazon, we discovered a new species of *Caenocentron*, the second known from South America and the first recorded from the Amazon ecoregion. In this study, we describe and illustrate this species and integrate it into the existing phylogenetic framework of the genus, offering new insights into the evolutionary history and biogeography of *Caenocentron*.

## 2. Materials and Methods

### 2.1. Study Area, Specimen Collection, Preparation, and Observation

The specimen was collected in the Savanna areas of Campos Amazônicos National Park, whose largest portion is located in the southern part of Amazonas State, Brazil [16] (Figure 1B). The collection was carried out using Malaise traps [17] (Figure 1C). The collected specimen was preserved in 96% ethanol. The holotype is deposited in the Coleção de Invertebrados, Instituto Nacional de Pesquisas da Amazônia (INPA), Manaus, Brazil.

To examine the male genital structures, the abdomen of each specimen was removed and cleared in hot 10% KOH, following the procedures described by Blahnik and Holzenthal [18]. After clearing, the abdomen was mounted on a temporary slide with glycerin and examined under a Leica DM5500 B compound microscope. Subsequently, it was stored in a microvial with glycerin, together with the remains of the respective specimen placed in a plastic vial containing 96% ethanol [19].

### 2.2. Illustrations and Map

Photographs of the habitus, head, and forewings were taken with a Leica DMC4500 digital video camera mounted on a Leica M205A stereo microscope or with a Leica DFC420 digital video camera mounted on a Leica M165C stereo microscope (Leica, Wetzlar, Germany). Multiple photographs of each structure were captured at different focal planes and subsequently stacked and combined into a single image using the Helicon Focus^®^ software (version Pro 7.6.4). Male genitalia were photographed with a Leica DFC295 video camera attached to a Leica DM5500 B compound microscope, and the stacked images were used as templates for vector graphic illustrations produced in Adobe Illustrator^®^, aided by a graphic tablet and pen (Intuos CTL4100, Wacom Technology Co., Saitama, Japan). All photographs and illustrations were further refined and arranged into plates using Adobe Photoshop^®^.

Distribution maps were generated using QGIS software (version 3.38.1-Grenoble), incorporating the terrestrial ecoregions shapefile from One Earth [20], based on the classification of Olson et al. [21]. Land cover and land-use were obtained from the “Projeto MapBiomas—Coleção 10 da Série Anual de Mapas de Cobertura e Uso da Terra do Brasil” [22], accessed through the MapBiomas Plugin in QGIS. For more information about the MapBiomas project, see Souza et al. [23]. Distribution data were compiled from literature sources and examined specimens.

### 2.3. Morphological Terminology and Description

Morphological terminology for the head setal warts follows Oláh and Johanson [24]. Male genitalia terminology is based on Nielsen [25] and Schmid [3], as interpreted for *Caenocentron* by Vilarino et al. [11]. Wing venation terminology follows the Comstock–Needham system, as applied to Trichoptera by Mosely and Kimmins [26]. To ensure consistency and standardization in descriptive taxonomy, the species description was generated using DELTA (Description Language for Taxonomy) software (version 1.02), based on a morphological matrix developed by Desidério et al. [27].

### 2.4. Phylogenetic Analysis

Phylogenetic inference was conducted using parsimony analysis based on the morphological dataset of Vilarino et al. [11], which now includes 26 taxa (16 ingroup and 10 outgroup taxa) and 46 morphological characters. The dataset matrix was edited using Mesquite software (version 3.7; Maddison and Maddison [28]). Analyses were performed in TNT (Tree analysis using New Technology; version 1.6; Goloboff and Morales [29]) under the following parameters: heuristic searches using “Traditional Search” with 10,000 tree bisection-reconnection (TBR) replications and five trees saved per replication. Implied weighting was applied using a rescaled concavity constant (*K* = 4.6), ensuring a minimum/maximum homoplasy weight ratio of 1:10, as implemented in TNT 1.6. Branch support was assessed via symmetric resampling (100 replications; Goloboff et al. [30]), expressed as the frequency difference in the CG (contradicted/present groups), and relative Bremer support (Goloboff and Farris [31]), with branch-swapping of suboptimal trees up to 10 steps longer and a relative fit of 0.9%. The resulting trees were visualized in Winclada (version 1.89; Nixon [32]), and the final figures were edited in Adobe Illustrator^®^. Unambiguous synapomorphies were mapped and displayed on the consensus tree using Winclada.

## 3. Results

### 3.1. Phylogenetic Placement

**Figure 2:**
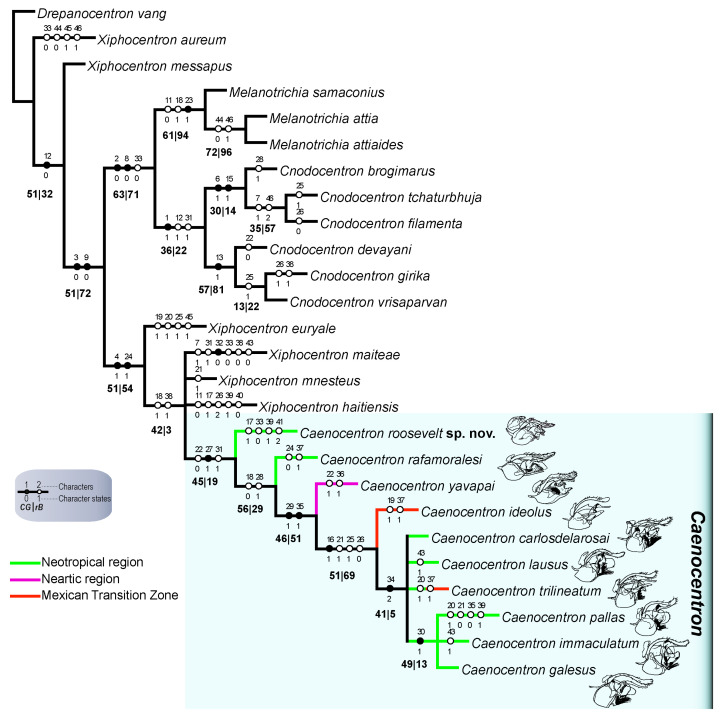
Strict consensus tree summarizing the six most parsimonious trees obtained under implied weighting (*K* = 4.6) for genera of Xiphocentronidae. Values in bold below branches represent support values: relative Bremer support [*rB*] (right) and symmetric resampling [*CG*] (left). Unambiguous character transformations are mapped along branches; black symbols indicate unique (non-homoplastic) character state changes.

The phylogenetic analysis under implied weighting (*K* = 4.6) yielded six most parsimonious trees, each with a length of 125 steps, a consistency index (CI) of 0.416, and a retention index (RI) of 0.714. The strict consensus tree is presented in Figure 2. In this topology, *Caenocentron roosevelt* sp. nov. was recovered as the earliest diverging lineage within the genus, positioned basally to *C. rafamoralesi* Vilarino, Dias & Bispo, 2022 from Costa Rica. This placement is supported by the following character states: 22(0)—preanal appendage slender; 27(1)—basal plate apodeme directed ventrad; and 31(1)—coxopodite apical margin produced posterad.

### 3.2. Taxonomy

#### 3.2.1. Species Description

**Figure 3:**
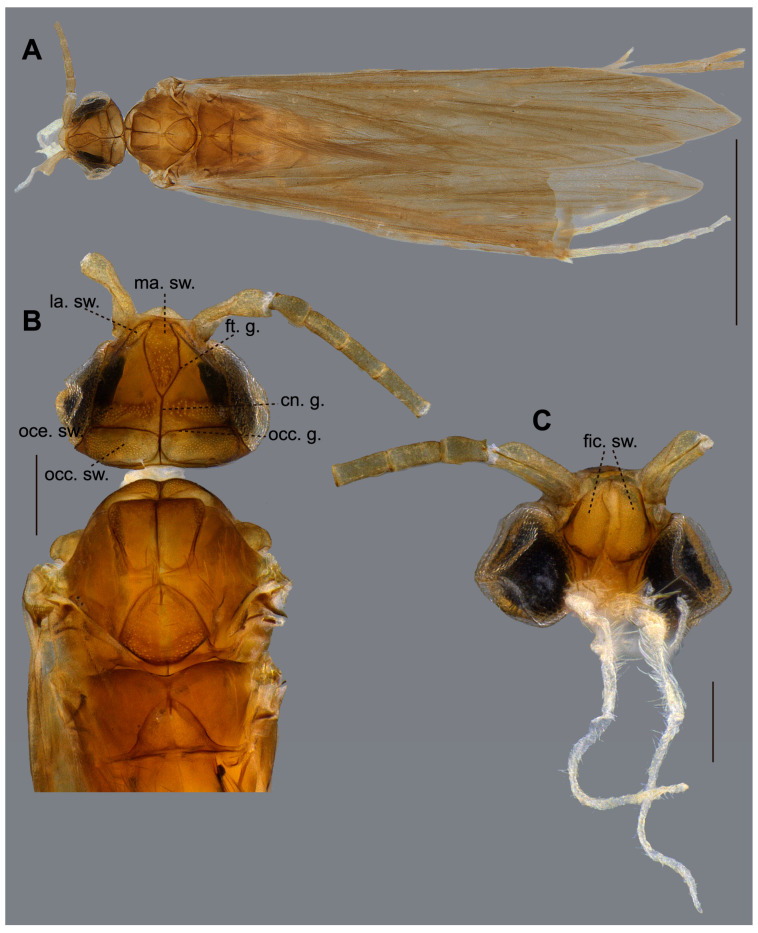
*Caenocentron roosevelt* sp. nov. (Xiphocentronidae), holotype male: (**A**) dorsal habitus; (**B**) head and thorax, dorsal view; (**C**) head, frontal view. Scale bars in mm: (**A**) 0.5; (**B**,**C**) 0.2.

**Figure 4:**
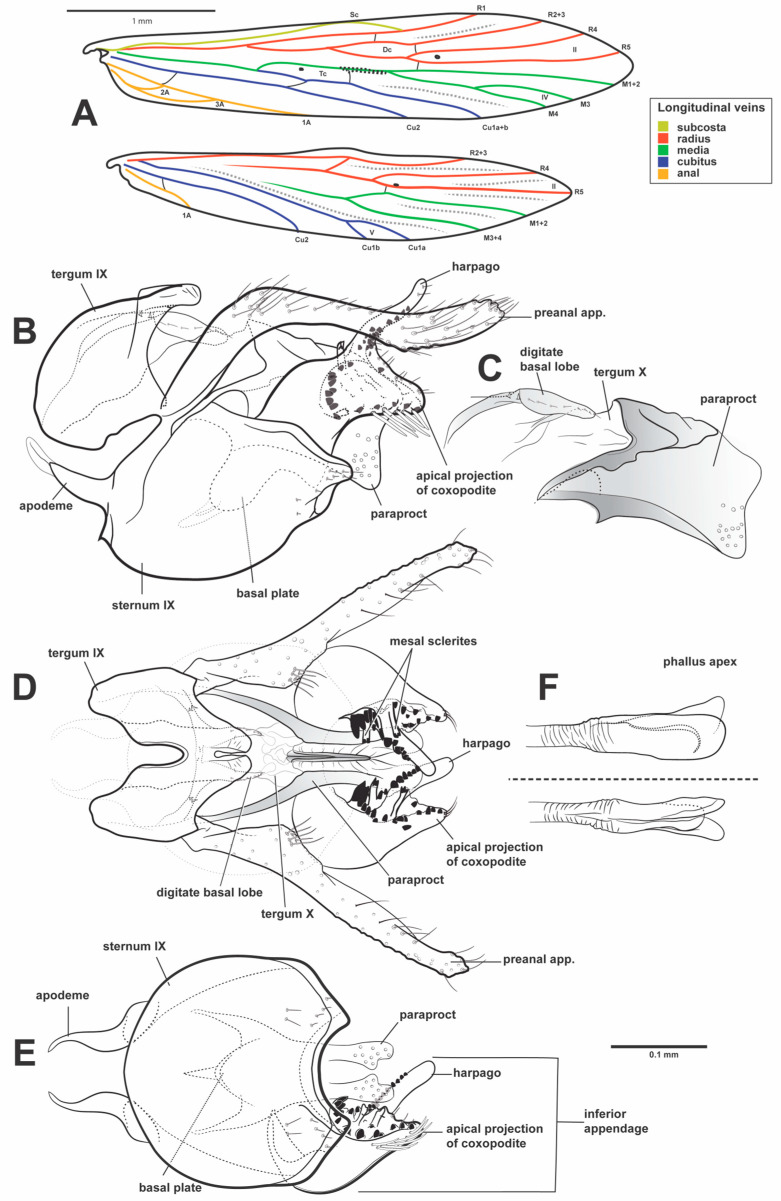
*Caenocentron roosevelt* sp. nov. (Xiphocentronidae), male genitalia (holotype): (**A**) venation of the right forewing (above) and hindwing (below), dorsal view (longitudinal veins highlighted in colors); (**B**) genitalia, left lateral view; (**C**) paraproct in detail, left lateral view; (**D**) genitalia, dorsal view; (**E**) genitalia, ventral view; (**F**) phallus, left lateral view (above), ventral view (below).

***Caenocentron roosevelt* sp. nov**.

urn:lsid:zoobank.org:act:A83F29D1-59EB-4682-8104-B6867797C3F8

(Figure 3 and Figure 4)

**Diagnosis.** *Caenocentron roosevelt* sp. nov. can be distinguished from its congeners by the following combination of characters: (1) presence of a distinct row of robust setae along the ventral margin of the protruding lobe of the inferior appendage; (2) absence of a setose ventral projection on the coxopodite; (3) presence of digitate lobes beneath the posterior margin of tergum IX; and (4) tergum IX elongate, with apical lobes distinctly developed.

**Description.** *Adult male*: Forewing length 3.74 mm (*n* = 1). General color pale brown (in alcohol) (Figure 3A,B). Head dorsally with seven setal warts; medioantennal setal wart (ma. sw.) large, reaching the mid-length of head, obovoid; lateroantennal pair (la. sw.) medium-sized, ellipsoid; ocellar pair (oce. sw.) large, ellipsoid; occipital pair (occ. sw.) very large, ellipsoid (Figure 3B); in frontal view, frontal interantennal pair (fic. sw.) large, fused mesally, each kidney-shaped (Figure 3C). Frontal grooves (ft. g.) V-shaped anteromesally, well-pronounced; coronal groove (cn. g.) thread-shaped, well-pronounced on posterior half of head; occipital groove (occ. g.) well-pronounced (Figure 3B). Maxillary palp segment length formula (I = II = III) < IV < V (Figure 3C). Forewing with forks II and IV; fork II sessile, without closed cell around nygma; discoidal cell about as long as thyridial cell. Hindwing with forks II and V; fork II sessile (Figure 4A). Tibial spur formula 2:4:3; spurs unmodified. Abdominal sternum V with reticulate anterolateral margin.

*Male genitalia*: Tergum IX, in lateral view, wider basally, narrower apically, about 1.5× as long as high (Figure 4B); in dorsal view, anterior margin with wide, very deep, Y-shaped mesal incision; posterior margin strongly produced posterad, divided apicomesally by deep, very narrow incision, with apex of tergite subtruncated (Figure 4D). Sternum IX, in lateral view, slightly longer than high, apex sinuous (Figure 4B); in ventral view, anterior margin convex, bearing a pair of apodeme about more than half of length this margin, narrow, sinuous, tapering to slender flange, and directed dorsad in lateral view; posterior margin produced posterad, divided apicomesally by deep, wide U-shaped mesal incision, with apex of sternite edges acute (Figure 4E). Tergum X membranous, fused to paraproct, bearing digitate basal lobe with short spines, and a row of setae to acute apex. Paraproct, in lateral view, oblong, with apex subtruncate, and bearing apicoventral margin produced ventrad (Figure 4C); in dorsal view, each side fused, with a sclerotized band on fused region, and sclerotized laterally; wide basally, tapering apically, divided apicomesally until sclerotized band, with numerous sensillae on semi-membranous apex (Figure 4D). Preanal appendage about 1.5× as long as tergum IX, setose; in lateral view, narrow, strongly curved at basal 1/3, wavy, narrower at midlength (Figure 4B); in dorsal view, slightly enlarged at basal 1/3 length (Figure 4D). Inferior appendage about as long as tergum IX; coxopodite and harpago completely fused; basal region wide, ventral margin highly prominent, rounded, bearing a row of long, stout spines, with inner surface bearing two slender mesal sclerites formed by long, stalk topped covered by small spines, and short spine-like spines distributed in curved line near mesal sclerites; apical region, in lateral view, wide at proximal portion, slender and digitate distally, about as long as basal region; inner surface with a row of numerous short, spine-like setae, more concentrated at base (Figure 4B,D); basal plate, in ventral view, triangular, reaching half the length of the sternum IX (Figure 4E). Phallus tubular, elongate, and slender, reaching segment V; basally conical, subapically annulate, weakly sclerotized, with apex slightly enlarged (Figure 4F).

**Distribution.** BRAZIL: Amazon biome (Amazonas state) (Figure 1).

**Material examined.** *HOLOTYPE MALE.* BRAZIL: Amazonas, Novo Aripuanã, Parque Nacional dos Campos Amazônicos, Igarapé da Onça (#04-AM); 8.6307° S, 61.431° W; 138 m a.s.l.; vii–viii.2017; N. Hamada, G.R. Desidério, P.V. Cruz, J.O. da Silva legs.; Malaise trap (INPA) (INPA-TRI 000000).

**Etymology.** The specific epithet is a homage to the Roosevelt River, formerly known as the “Rio da Dúvida” (River of Doubt), located in Brazil and originating in the state of Rondônia. The name honors former U.S. President Theodore Roosevelt, who played a key role in the Rondon-Roosevelt Scientific Expedition (1913–1914), which aimed to determine whether the river was a tributary of the Amazon. The epithet is used in apposition.

**Remarks.** The produced, rounded apical margin of the coxopodite in the inferior appendage observed in *C. roosevelt* sp. nov. is shared with some other species of *Caenocentron*, including *C. galesus* Schmid, 1982 (Costa Rica and Panama) and *C. pallas* Schmid, 1982 (Panama), as well as with species of the Oriental genus *Cnodocentron* and the Neotropical genus *Xiphocentron*. In the Brazilian *Xiphocentron*, this structure is notably present in *X. maiteae* Vilarino & Calor, 2015, *X. copacabana* Vilarino, Cavalcante, Dumas & Nessimian, 2018, and *X. muelleri* Vilarino & Bispo, 2020, highlighting the unresolved and potentially confusing relationships within *Xiphocentron* and among xiphocentronid genera. However, *C. roosevelt* sp. nov. can be readily distinguished from all other species of *Caenocentron* by a combination of characters: (1) the absence of a setose ventral projection on the coxopodite, a feature variably present in all other congeners; (2) the elongate tergum IX with distinct apical lobes, which is a feature similar to certain *Xiphocentron*, while *Caenocentron* show reduced tergum IX; and (3) the presence of digitate lobes near the posterior margin of tergum IX, a rare condition in Neotropical xiphocentronids, though a similar structure is reported in *C. yavapai* Moulton & Stewart, 1997 from the southern United States.

#### 3.2.2. Identification Key to Adult Male of Caenocentron (Modified from Vilarino et al. [11])

1 Sternum IX with apical margin produced into elongate processes ……………..……. 2

- Sternum IX with apical margin not produced, concave ……….……..….….………… 3

2 Apical projection of sternum IX forming two narrow, elongate processes ………………………………………………………………………………... *C. rafamoralesi*

- Apical projection of sternum IX forming a broad, deltoid plate with two short apical processes ………………………………………………..…………....................... *C. yavapai*

3 Coxopodite apical margin projection narrow, acute …………………….….…………. 4

- Coxopodite apical margin projection broad …………………………………..……….. 5

4 Sternum IX in lateral view truncate apically; coxopodite in ventral view with ventral projection bearing long, dense setae along most of the inner margin, including mesal region; each coxopodite with one short subapical spine on inner face … *C. trilineatum*

- Sternum IX in lateral view deltoid apically; coxopodite in ventral view with ventral projection bearing lateral setal brushes, mesal region glabrous; each coxopodite with two short spines on inner face …………………...……..………………………. *C. ideolus*

5 Coxopodite apical margin with a short spine or multiple long spine-like setae...….. 6

- Coxopodite apical margin lacking spines ………………………………...……………. 9

6 Coxopodites broadly fused at base in ventral view; inner margin with brush of long setae; apical margin bearing a short spine ………………………………….………...… 7

- Coxopodites separate at base in ventral view; inner margin without setal brush; apical margin with line of several long spine-like setae …………….. *C. roosevelt* sp. nov.

7 Coxopodite inner margin (ventral view) with a pair of long sublateral stout spines; basal third of harpago strongly enlarged …….………………….……... *C. immaculatum*

- Coxopodite inner margin (ventral view) lacking stout sublateral spines; basal third of harpago slightly or not enlarged ……………………………………….……………. 8

8 Harpago base with linear setal brush; coxopodite apical margin projection broad to apex, bearing short ventroapical spine …………………….…………………… *C. lausus*

- Harpago base lacking setal brush; coxopodite apical margin projection tapering to rounded apex, bearing a subapical short spine ………………………. *C. carlosdelarosai*

9 Inferior appendage with lobe bearing brush of setae near harpago base; coxopodite median region with nearly indiscernible small setules; paraproct dorsal margin without process, ventroapical margin rounded …………...…………………..……... *C. pallas*

- Inferior appendage without brush of setae near harpago base; coxopodite median region with transverse patch of setae; paraproct dorsal margin with short, acute process, ventroapical margin acute…………………………………………………. *C. galesus*

## 4. Discussion

New World Xiphocentronidae are hypothesized to have originated from an Oriental ancestor that crossed the Bering Strait during hyperthermal periods [3,11]. Within this framework, *Caenocentron* was considered to have evolved from a *Xiphocentron*-like ancestor in Mesoamerica, where it initially diversified before dispersing into South America following the closure of the Central American Seaway during the late Miocene. However, the phylogenetic placement of *C. roosevelt* sp. nov. as the sister taxon to all other *Caenocentron* species considerably alters this biogeographic narrative. If this placement is correct, it implies that *Caenocentron* reached South America significantly earlier than previously proposed. Based on the time-calibrated phylogeny, the earliest cladogenesis after *C. roosevelt* sp. nov. occurred around 26 million years ago [11], suggesting that the genus was present in South America prior to that time.

One plausible explanation for this earlier arrival is the Gaarlandia land-bridge hypothesis, which proposes a connection between northern South America and the Greater Antilles via the Aves Ridge around 35–32 Ma [33,34]. This scenario supports the possibility of an early colonization of South America by ancestral *Caenocentron*, potentially challenging the assumption that the genus originated in Mesoamerica. Instead, these findings raise the alternative hypothesis that *Caenocentron* may have originated in South America, with subsequent northward dispersal.

The Amazon ecoregion has long been considered poorly represented in terms of Xiphocentronidae diversity, with only eight species previously recorded: *Xiphocentron* (*Antillotrichia*) *sclerothrix* Pes & Hamada, 2013 and *Machairocentron falciforme* Pes & Hamada, 2013 from Brazil; *X*. (*Antillotrichia*) *ashaninka*, *X*. (*A.*) *harakbut*, *X*. (*A.*) *matsigenka*, *X*. (*A.*) *yine*, and *M. amahuaca* Vilarino, Salles & Bispo, 2023 from Peru; and *X*. (*A.*) *surinamense* Flint, 1974 from Suriname [6,12,35]. However, current evidence suggests that this view significantly underestimates the true diversity of Xiphocentronidae in the region. Desidério et al. [27] recently expanded the known diversity by describing three new species and providing new distribution records for *M. amahuaca*, *M. falciforme*, *X*. (*A.*) *sclerothrix*, and *X*. (*A.*) *surinamense* from remote areas of the Brazilian Amazon.

Additional results from targeted taxonomic research and focused sampling efforts, particularly within Campos Amazônicos National Park [36], led to the discovery of *C. roosevelt* sp. nov.—the second species of *Caenocentron* recorded in South America and the first from the Amazon ecoregion. This finding highlights the hidden diversity of caddisflies in western Amazonia, one of the least-explored regions of the Neotropics. The region presents significant logistical challenges, requiring substantial infrastructure and funding to support sustained research efforts [37].

Another highly relevant aspect of the discovery of *C. roosevelt* sp. nov. is that its type locality lies within a strictly protected conservation unit, Campos Amazônicos National Park. This underscores the importance of expanding protected areas in Brazil, which covers approximately 25% of the national territory and safeguards about 40% of the remaining native vegetation, primarily within the Amazon [38,39,40]. Campos Amazônicos National Park constitutes the largest refugium of savanna formations in the southern Brazilian Amazon, characterized predominantly by herbaceous and shrubby vegetation (Figure 1B) [16]. Nevertheless, the region faces significant anthropogenic pressure, as the agricultural frontier continues to expand, and forested areas adjacent to the park have already suffered extensive deforestation for farming (Figure 1B) [22]. Consequently, both the extensive hydrographic network and the organisms dependent on it are increasingly threatened. Future studies should therefore prioritize under-sampled areas such as this, which harbor high potential for the discovery of new taxa and for advancing our understanding of Xiphocentronidae diversity and biogeography in the Neotropical region.

## 5. Conclusions

In this study, we describe *C. roosevelt* sp. nov., the first species of the genus recorded from the Amazon ecoregion. Phylogenetic analysis recovered this species as the earliest diverging lineage within *Caenocentron*, supported by a combination of morphological characters: a slender preanal appendage, the basal plate apodeme directed ventrally, and the coxopodite apical margin produced posteriorly. The placement of *C. roosevelt* sp. nov. at the base of the genus significantly alters our understanding of the evolutionary history and biogeography of *Caenocentron*. Rather than a relatively recent (late Miocene) dispersal into South America from Mesoamerica, as previously inferred, our results suggest a much earlier colonization—potentially during the Oligocene or earlier. This raises the possibility that *Caenocentron* may have originated in South America, challenging earlier hypotheses of a strictly Mesoamerican origin.

## Data Availability

All available data are presented in the present study, and specimens are vouchered as indicated in the examined material Section 3.2.1.

## Abbreviations

The following abbreviations are used in this manuscript:

cn. g	coronal groove
fic. sw.	frontal wart
ft. g.	frontal grooves
la. sw.	lateroantennal wart
ma. sw.	medioantennal wart
occ. g.	occipital groove
occ. sw.	occipital wart
oce. sw.	ocellar wart
